# Linking stress with urocortin in rats

**DOI:** 10.6026/973206300191057

**Published:** 2023-11-30

**Authors:** Manikandan Balraj, Ankamma Sarvepalli, Bijoya Chatterjee, Gnanadesigan Ekambaram, Nithya Rajapandian, K Nisha, Vadivel Mani

**Affiliations:** 1 Department of Physiology, Konaseema Institute Medical Science and Research Foundation, Amalapuram, East Gothawri - 533201, Andhra Pradesh, India; 2 Department of Anatomy, Konaseema Institute Medical Science and Research Foundation, Amalapuram, East Gothawri - 533201, Andhra Pradesh, India; 3 Department of Biochemistry, M. P Shah Government Medical College , Jamnagar - 361008 Gujarat, India; 4 Department of Physiology, Nootan Medical College and Research Center, Sankalchand Patel University, Visnagar - 384315, Gujarat, India; 5 Medical physiology, Mahatma Gandhi Medical College and Research Institute, Sri Balaji Vidyapeeth, Puducherry - 607402, India; 6 Department of Community Health Nursing, KIMS Nursing College, KIMS&RF Amalapuram, East Gothwari - 533201, Andhra Pradesh, India; 7 Department of Biochemistry, Konaseema Institute Medical Science and Research Foundation, Amalapuram, East Gothawri - 533201, Andhra Pradesh, India

**Keywords:** Chronic variable stress, urocortin, corticotrophin-releasing factor, HPA-axis

## Abstract

The corticotropin-releasing factor neuropeptides (CRH and UCN-1,2,3), as well as spexin, contribute to the control of energy balance and limit food intake in mammals. However, the role of these neuropeptides in chronic variable stress remains unknown. The effect of chronic varied stress on circulating corticosterone levels and urocortin expression levels in the brains of experimental rats was studied in this study. Rats were subjected with 28 days long term stress protocol, end of stress protocol experimental and control animal organs isolated, brain urocorcortin-1,2,3 expression by RT-PCR and serum corticosterone by ELISA method. UCN levels in the brain were altered in rats subjected to prolonged varied stress. Furthermore, corticosterone levels were elevated as a result of the same urocortin expression pattern, indicating that urocortin expression is controlled by glucocorticoids via a glucocorticoid-responsive element (GRE). Thus, data shows that hypothalamus-pituitary-adrenal (HPA) axis, also known as the LHPA axis, and limbic system are both stimulated by stress, which is reflected in the form of elevated corticosterone levels, according to the genes UCN1, 2, and 3.

## Background:

Stress is frequently described as an external input that upsets an organism's physiological balance [1]. The degree of the stimulus, along with an individual's capacity to adapt on a behavioural, physiological, and molecular level, determines whether stress results in adaptive or maladaptive outcomes [2]. The duration of stress is also very important for its pathogenic effects, in addition to the stressor's degree [3]. Chronic stress exposure causes a variety of harmful effects on human health, including nervous system problems including neurodegenerative disease, mood disorders, and systemic diseases like cardiovascular disease, metabolic conditions [4]. The brain is a significant stress target, and it adapts to psychological or physical stressors by changing its emotions, behaviours, cells, and molecules [5]. When phrases like "long-term" and "chronic" are used to characterize stressors and their effects, stress duration and persistence of stress effects must be taken into account separately [6]. This susceptibility to chronic stress-related disease in rodents has been successfully duplicated by the widely used chronic variable stress (CVS) paradigm, most notably through the creation of a depressive-like behavioural state [7]. Upon stress exposure, CRF is rapidly released from the paraventricular nucleus (PVN) of the hypothalamus into the periphery to activate the hypothalamic-pituitary-adrenal (HPA) axis by stimulating the release of adrenocorticotropic hormone from the anterior pituitary, which then triggers the synthesis and secretion of corticosteroids (cortisol in humans, corticosterone in rodents) from the adrenal gland [8]. Urocortins (Ucns), peptides belonging to the corticotropin-releasing hormone (CRH) family, are classified into Ucn1, Ucn2, and Ucn3. They are involved in regulating several body functions by binding to two G protein-coupled receptors: receptor type 1 (CRHR1) and type 2 (CRHR2) [9]. Therefore, it is of interest to find out how Wistar rats' urocortin expression was affected by chronic varied stress.

## Materials and methods:

## Study design:

This study was conducted in the Department of Physiology, Meenakshi Medical College Hospital & Research Institute, Enathur, Kanchipuram. All the experiments were carried out according to the protocol approved by the Institutional Animal Ethical Committee (CPCSEA) (KN/COL/3411/2014). All experiments were carried out on adult male Wistar albino rats with weight ranging from 120-150 grams. Animals were housed under a 12 hrs light / dark cycle in a room with controlled temperature (23±2°C) and with free access to food and water. Twenty-four animals were taken for our studies, which was divided into four groups, which contain six animals in each group. The grouping of animals is shown in Table 1

## Stress techniques:

The Chronic variable stress (CVS) model used was a modified version of chronic variable stress procedure used in previous experiments [10]. Different stressors with different duration were applied one by one every day. Each morning, all animals were tail marked with coloured markers to ensure proper identification. Stressors were administered in a different room than the conditioning and testing rooms and all transportation to and from the various labs was done with wheeled laboratory carts. During these 28 days, Control Group animals were not exposed to chronic variable stress and remained in their home-cages. Furthermore, Control Group animals received the same transportation exposure as the Experimental Group animals, to ensure that any stress from the act of transportation was controlled.

Stressors were administered from 9:00 to 16:00 to avoid variability due to circadian rhythms, and all measurements were carried out during the light phase between 9:00 and 13:00. The chronic stress protocol and timing are described in Table 2.

Two days after the stress treatment, sodium thiopental (40 mg/kg b.w.t.) was used to sedate Control and experimental animals and draw blood through cardiac puncture. 20 ml of isotonic sodium chloride (NaCl) solution was perfused through the left ventricle to remove blood from the organs, and serum was isolated and maintained at -80°C. To measure the expression of the urocortin gene, the brain was dissected.

## Assessment of Serum corticosterone:

Plasma samples were used for analysis of hormone levels in duplicate using ELISA kits. Corticosterone was measured using kit K014-H1 (Arbor Assays, Ann Arbor, MI USA). Hormone analysis was done with the instructions of the manufacturers.

## mRNA expression analysis:

## Total RNA Isolation, cDNA conversion and real-time PCR

A TRIR kit (Total RNA Isolation Reagent Invitrogen) was used to extract total RNA from the control and experimental samples. In a nutshell, 100 mg of fresh tissue received 1 ml of TRIR, which was then homogenized. The material was then immediately transferred to a micro centrifuge tube, combined with 0.2 ml of chloroform, vortexed for 1 minute, and stored at 4°C for 5 minutes. Then, the mixture was centrifuged at 12,000 g for 15 minutes at 4 °C. Carefully transferring the top layer of the aqueous phase to a fresh microfuge tube, equal parts of isopropyl alcohol were then added, vortex for 15 seconds, and then placed on ice for 10 minutes. Following centrifugation of the material at 12000g for 10 minutes at 4C, the supernatant was separated. The RNA pellet was washed in 1 ml of 75% ethanol using the vortex. The extracted RNA was calculated using spectrometry according to Fourney et al. Each sample's RNA content was quantified in micrograms.

Using a reverse transcriptase kit from Eurogentec (Seraing, Belgium), complementary DNA (cDNA) was created from 2 micrograms of total RNA in accordance with the manufacturer's instructions. A 45 µl reaction mixture containing 2x reaction buffer (Takara SyBr green master mix), forward and reverse primers for the target and housekeeping genes, water, and β-actin (primer sequences are supplied in (Table 3) was made in order to perform real-time PCR. About 5 µl of control DNA for the positive control, 5 µl of water for the negative control, and 5 µl of template cDNA for the samples were extracted and added to each individual PCR vial along with the reaction mixture (45 µl). The reaction was set up for 40 cycles (95°C for 5 min, 95°C for 5 s, 60°C for 20 s, and 72°C for 40 s), and the PCR machine (Stratagene MX 3000P, Agilent Technologies, 530l, Stevens Creek Blvd, Santa Clara, CA, 95051) showed the findings on a graph. From the examination of the melt and amplification curves, relative quantification was derived.

## Statistical analysis:

Using one-way analysis of variance (ANOVA) and Duncan's multiple range test; computer-based software, the data were analyzed to determine the significance of individual variance within the control and treated groups (Graph Pad Prism version 5). Duncan's test was used to determine significance at the level of p<0.05.

## Results:

## Chronic variable stress induces HP axis mediated stress response:

**Figure 1:**
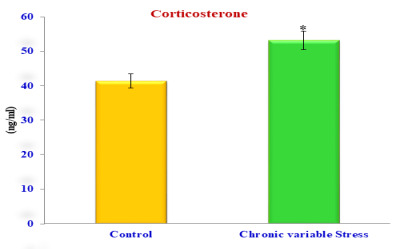
Serum corticosterone level of control and chronic variable stress induced rats. Values are expressed as ng/ml. Each value represents the mean ± S.E. obtained from six different experiments. *P<0.05 vs control.

Exposure of rats to chronic variable stress for the experimental period, at the end of the study the hormonal assay was performed from the serum of control and chronic stress induced rats. The resulted in a significant elevation in the level of serum corticosterone compared to the control animals (Table 4 and Figure 1). Corticosterone was significantly higher in rats exposed to chronic variable stress compared to its level in control rats. Serum corticosterone levels were p <0.05 significant to control rats.

##  Chronic variable stress increases Urocortin1 (Ucn1) mRNA expression in the brain of experimental rats:

UCN1, UCN2, and UCN3 mRNA expression in the stress group significantly increases in molecular studies. In RT-PCR, the amplicon's accumulation and amount of fluorescence are monitored during the reaction. The amplification reaction's fluorescence, which is proportional to the amplified sample for each cycle of the reaction, is plotted. The reaction's cycle count is shown on the X-axis, while the amplicon's fluorescence rate is shown on the Y-axis.

**Figure 2:**
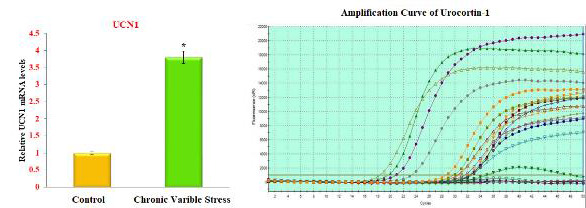
Expression of Urocortin-1 level in brain of control and experimental rats

**Figure 3:**
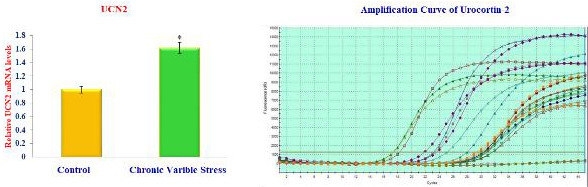
Expression of Urocortin-21 level in brain of control and experimental rats.

**Figure 4:**
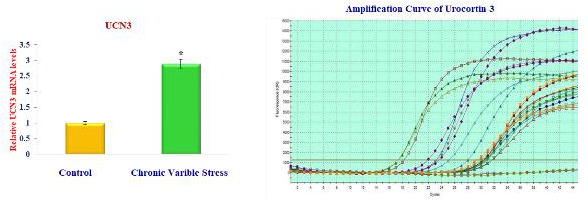
Expression of Urocortin-3 level in brain of control and experimental rats.

At the end of the 34th cycle, the urocortin-1, urocortin-2, and urocortin-3 amplification graph (Figure 2, Figure 3, and Figure 4) shows peaks at various frequencies. The standard samples used as controls are shown by the two peaks in green and yellow that are present below the threshold frequency line. At the conclusion of the 34th cycle, the peak in purple and dark green representing the dark stressed group samples displayed the highest fluorescence levels. The purple shade's curve, on the other hand, has the strongest fluorescence and deviates from the norm with greater frequency. This exhibits enhanced expression of the Urocortin-1,2,3 variation from the conventional sequence and has the largest concentration of the amplicon in the 34th cycle.

## Discussion:

A possible explanation for the higher corticosterone levels in this study is HPA axis activation, more especially stimulation of the paraventricular nucleus in the hypothalamus. Additionally, it plays a role in the sympathetic nervous system's activation, which raises the levels of serotonin and tryptophan in the brain in response to stress. These observations support the conclusions made by Silverman et al. 2005 [15]. Corticosterone regulates both the expression of the UCN1 gene in the Edinger-Westphal nucleus (EW) and the expression of the hypothalamic CRF throughout the stress adaption process [16]. More specifically, glucocorticoids, and particularly corticosterone, also control CRF and Ucn2 as well as other peptides belonging to the CRF family [17]. The existence of GR in CRF-expressing neurons shows that glucocorticoids have an immediate impact on these neurons.

The sequence homology between the urocortins, UCN1, UCN2, and UCN3, and the corticotropin releasing factor (CRF), sauvagine, and urotensin 1 led to their discovery [18]. In the current investigation, the chronic variable stress-induced group of rats had considerably higher levels of Urocortin-1, Urocortin-2, and Urocortin-3 gene expression than the healthy control rats. Urocortin-1 is a key gene in stress adaptation, according to Bayan et al (2013), and this finding is supported by Kozicz et al.'s (2021) observation that UCN

1 mRNA is upregulated in brain samples from depressed men, suicide victims, etc., which may be related to the role of UCN1/CRF2 in long-term adaptation and recovery from stress [19,20,21]. In our investigation, UCN2 revealed a large increase in expression, which may be related to a spike in neurotransmitter levels induced by stress and moderated by the hormone corticosterone, which may result in pathological changes in the brain. This is a crucial element enabling UCN2's increased expression during chronic varied stress. These findings support the conclusions reached by recent research findings [22]. It is completely unknown what functions Ucn-3 might have in reactions to physiological situations. Thus, we have examined the effects of chronic variable stress on UCN-3 expression in the brain and hormonal aspects of the stress response. UCN3 showed up-regulated expression which could be due to the presence of the neurons, which originated from medial amygdala, the hypothalamic median preoptic nucleus, and the rostral perifornical area lateral to the paraventricular nucleus [23]. According to Kuperman Yael *et al*. (2010) research chronic irreversible stimulation of neurons generates pathophysiological alterations such mood disturbance and behavioural abnormalities via the neuromodulator Urocortin-3 in chronic stress-induced rats, this observation supports our study results [24].

Rats subjected to chronic variable stress showed increase in UCN 1,2 & 3 might be a pathway dependent effect which static that psychogenic stressor through limbic circuits relay on PVN which causes up-regulated of glutamatergic tone which alters the HPA - axis. Stressor stimulate the profused production of glucocorticoids in the adrenal cortex which influence HPA axis, but in two ways like negative feedback effect on hypothalamic CRF and positive feedback effect on amygdala CRF [25]. In addition, amygdala CRF further stimulate the hypothalamic CRF through "Feed - Forward" effect and this influence highest production of CRF and hence increased expression of UCN 1, 2 & 3. Increased Urocortin"s after chronic variable stress is in accordance with the findings of [26, 27] where stress induces neurodegenerative process with in the limbic system and hippocampus which causes more detrimental effects by high production of glucocorticoids which leads to the production of free radicals in the neurons because of oxidative stress [26, 27]. It has been postulated that glucocorticoids regulate CRF transcription via a cAMP-responsive element (CRE) present in the promoter of the CRF-gene [28] Since CRE is also present in the promoter of the mouse UCN1 gene it might be a target for glucocorticoids to regulate UCN1-gene expression [29]. On the other hand, the expression of UCN2 in the mouse hypothalamus and brainstem appears to be controlled by glucocorticoids via a glucocorticoid-responsive element (GRE) in the Ucn2-gene promoter were reported [30, 31]. The role of CRH in the hypothalamic pituitary adrenal (HPA) axis stress response is well documented [32]. The activation of HPA axis together with the increased expression of corticosterone level because of activation of the sympatho-adreno-medullary system, are the key regulators of the organism's chronic stress response [33]. Our study reveals that chronic variable stress induced rats showed significantly increased expression of UCN1 & UCN2 mRNA level; this supports the increased expression of corticosterone on the HPA axis.

## Conclusion:

Data shows that modifying the genetic alterations of Urocortin resulted in a higher stressor by demonstrating chronic variable stress. Using a highly standardized RT-PCR technique, the expression of the CRF family genes UCN1, 2, and 3 was used to evaluate the impact of chronic varied stress in great detail. The hypothalamus-pituitary-adrenal (HPA) axis, also known as the LHPA axis, and limbic system are both stimulated by stress, which is reflected in the form of elevated corticosterone levels, according to the genes UCN1, 2, and 3.

## Funding sources:

No funding was received for this research.

## Figures and Tables

**Table 1:** Study design

Group	Animals	Numberofanimals
Group1	A	B
Group2	1.18	0.94

**Table 2:** Chronic stress protocol and timing

Day	Stress treatment
1	1hr restraint, alone
2	2 hr in new cage with wet bedding
3	Alone, Forced swim test, 5mins
4	Tail pinches in restrainer, 20 mins
5	Cold water swim test, 5mis
6	Crowding, overnight
7	Isolation, overnight
8	1hr restraint, alone
9	2 hr in new cage with wet bedding
10	Alone, Forced swim test, 5mins
11	Tail pinches in restrainer, 20 mins
12	Cold water swim test, 5mis
13	Crowding, overnight
14	Isolation, overnight
15	1hr restraint, alone
16	2 hr in new cage with wet bedding
17	Alone, Forced swim test, 5mins
18	Tail pinches in restrainer, 20 mins
19	Cold water swim test, 5mis
20	Crowding, overnight
21	Isolation, overnight
22	1hr restraint, alone
23	2 hr in new cage with wet bedding
24	Alone, Forced swim test, 5mins
25	Tail pinches in restrainer, 20 mins
26	Cold water swim test, 5mis
27	Crowding, overnight
28	Isolation, overnight

**Table 3:** Primer sequences of Urocortin molecules

Name of the gene	Primer Sequence	Reference
Ucn1[11]	Sense primer: -5'- TATAGATCTGGCACCATGAGGCAG AGGGGA-3' Anti-sense primer: - 5'CGCGAATTCCGATCACTTGCCCACC GAATC-3'	Chang J*et al*,2021
Ucn2[12]	Sense primer: 5'-TGGGCACTGGTGGTGTTTATGG-3' Anti-sense primer: 5'-CCAGAACTTCTCATCCAGGGTCAC-3'	EM Fekete*et al*2007
Ucn3[13]	Sense primer: 5'- CGAAGTCCCTCTCACACCTGGTT-3' Anti-sense primer: 5'- CGGCAAACGGACAGAAGCATT -3'	Deyana*et al*2021
Rat β-actin[14]	Sense primer: 5'- AAG TCC CTC ACC CTC CCA AAA G-3' Anti-sense primer: 5'- AAG CAA TGC TGT CAC CTT CCC-3'	Peinnequin*et al*2004

**Table 4:** Serum corticosterone levels in control and experimental rats

HP axis mediated stress response	Control	Chronic variable stress
Corticosterone ng/ml	41.35±1.01	53.19±2.31
HP axis mediated stress response	Control	Chronic variable stress
Corticosterone ng/ml	41.35±1.01	53.19±2.31
HP axis mediated stress response	Control	Chronic variable stress
Values are expressed as ng/ml. Each value represents the mean ± S.E. obtained from six different experiments. *P<0.05 vs control.
